# Physicochemical and biological factors controlling water column metabolism in Sundarbans estuary, India

**DOI:** 10.1186/2046-9063-8-26

**Published:** 2012-10-19

**Authors:** Kaberi Chaudhuri, Suman Manna, Kakoli Sen Sarma, Pankaj Naskar, Somenath Bhattacharyya, Maitree Bhattacharyya

**Affiliations:** 1 Institute of Environmental Studies and Wetland Management, DD-24, Sector-I, Salt lake, Kolkata, 700064, India; 2 Department of Biochemistry, University of Calcutta, 35, Ballygunge Circular Road, Kolkata, 700019, India

**Keywords:** Net ecosystem metabolism, Gross primary productivity, Community respiration, Nitrification, Nutrient load, Sundarban estuary

## Abstract

**Background:**

Sundarbans is the single largest deltaic mangrove forest in the world, formed at estuarine phase of the Ganges - Brahmaputra river system. Primary productivity of marine and coastal phytoplankton contributes to 15% of global oceanic production. But unfortunately estuarine dynamics of tropical and subtropical estuaries have not yet received proper attention in spite of the fact that they experience considerable anthropogenic interventions and a baseline data is required for any future comparison. This study is an endeavor to this end to estimate the primary productivity (gross and net), community respiration and nitrification rates in different rivers and tidal creeks around Jharkhali island, a part of Sundarbans estuary surrounded by the mangrove forest during a period of three years starting from November’08 to October’11.

**Results:**

Various physical and chemical parameters of water column like pH, temperature, conductivity, dissolved oxygen, turbidity, suspended particulate matter, secchi disc index, tidal fluctuation and tidal current velocity, standing crop and nutrients were measured along with water column productivity. Relationship of net water column productivity with algal biomass (standing crop), nutrient loading and turbidity were determined experimentally. Correlations of bacterial abundance with community respiration and nitrification rates were also explored. Annual integrated phytoplankton production rate of this tidal estuary was estimated to be 151.07 gC m^-2^ y^-1^. Gross primary productivity showed marked inter annual variation being lowest in monsoon and highest in postmonsoon period.

**Conclusion:**

Average primary production was a function of nutrient loading and light penetration in the water column. High aquatic turbidity, conductivity and suspended particulate matter were the limiting factors to attenuate light penetration with negative influence on primary production. Community respiration and nitrification rates of the estuary were influenced by the bacterial abundance. The estuary was phosphorus limited in postmonsoon whereas nitrogen-limited in premonsoon and monsoon period. High algal biomass and primary productivity indicated the estuary to be in eutrophic state in most of the time throughout the year. Our study also indicated a seasonal shifting between autotrophic and heterotrophic conditions in Sundarban estuarine ecosystem and it is a tropical, well mixed (high tidal influx) and marine dominated (no fresh water connection) system.

## Background

Primary productivity of marine and coastal phytoplankton contributes to 15% of global oceanic production. Phytoplankton biomass and primary production mainly depend on nutrient dynamics of coastal and estuarine ecosystems [1,2]. Anthropogenic inputs of excess nutrients to coastal waters is a global problem and has increased dramatically over last few decades [1,3,4].These interventions affect phytoplankton dynamics and primary productions to a great extent [5]. On the other hand enhanced nutrient loading also stimulates primary production [2,3]. Phytoplankton are known for their rapid responses to altered environmental conditions [6], such as anthropogenically introduced eutrophication of coastal waters [7], alteration of coastal configuration, degradation of environmental conditions e.g. sewage outfalls with the consequent water pollution [8,9] etc. The dynamic structure of phytoplankton communities directly reflects the health of aquatic ecosystems [10]. In India, Ganges-Brahmaputra estuary is particularly vulnerable to anthropogenic perturbations due to high nutrient loads from riverine discharge, increasing human population density and rapid economic growth [11,12]. In Indian Sundarbans a huge quantity of leaf litter is loaded to the estuarine water from surrounding mangrove forests. Land mass washes during monsoon and effluents from shrimp culture farms also contribute to this huge nutrient load.

Nutrient enrichment or loading has already been identified as a serious problem in recent times in many estuaries [13] in the world namely Baltic sea, Adriatic sea, Gulf of Maxico, Chesapeake Bay and San Francisco Bay especially during rapid growth of population, agriculture and fertilizer production [1,2]. Rates and patterns of nutrient assimilation vary widely among the coastal ecosystems all over the world. While some estuaries exhibits acute eutrophication leading to enhanced algal biomass and primary production, other nutrient rich estuaries maintain low algal biomass and primary production [2]. Estuarine dynamics has been well studied in temperate system such as Chesapeake Bay [14,15], San Francisco Bay [16], and the Baltic sea [17]. Tropical and subtropical estuaries received comparatively less study but are experiencing noticeable anthropogenic alterations [18,19].

Estuaries are highly dynamic systems linking land to the ocean with large seasonal and spatial gradients of biogeochemical compounds and processes [20]. They are often greatly influenced by anthropogenic activities, including enhanced organic matter and nutrient loadings. Net ecosystem metabolism (NEM), the net effect of production and respiration, in coastal and estuarine environments is a means to evaluate whether such environments are sources or sinks of carbon [21,22]. NEM is positive (autotrophic) when production exceeds respiration and negative (heterotrophic) when respiration exceeds production. Most estuaries are net heterotrophic [21,22] and generally a net source of CO_2_ to the atmosphere [23-25]. The balance between organic matter and nutrient loading is critical in determining the balance between autotrophy and heterotrophy at the ecosystem level [26,27].

**Figure 1:**
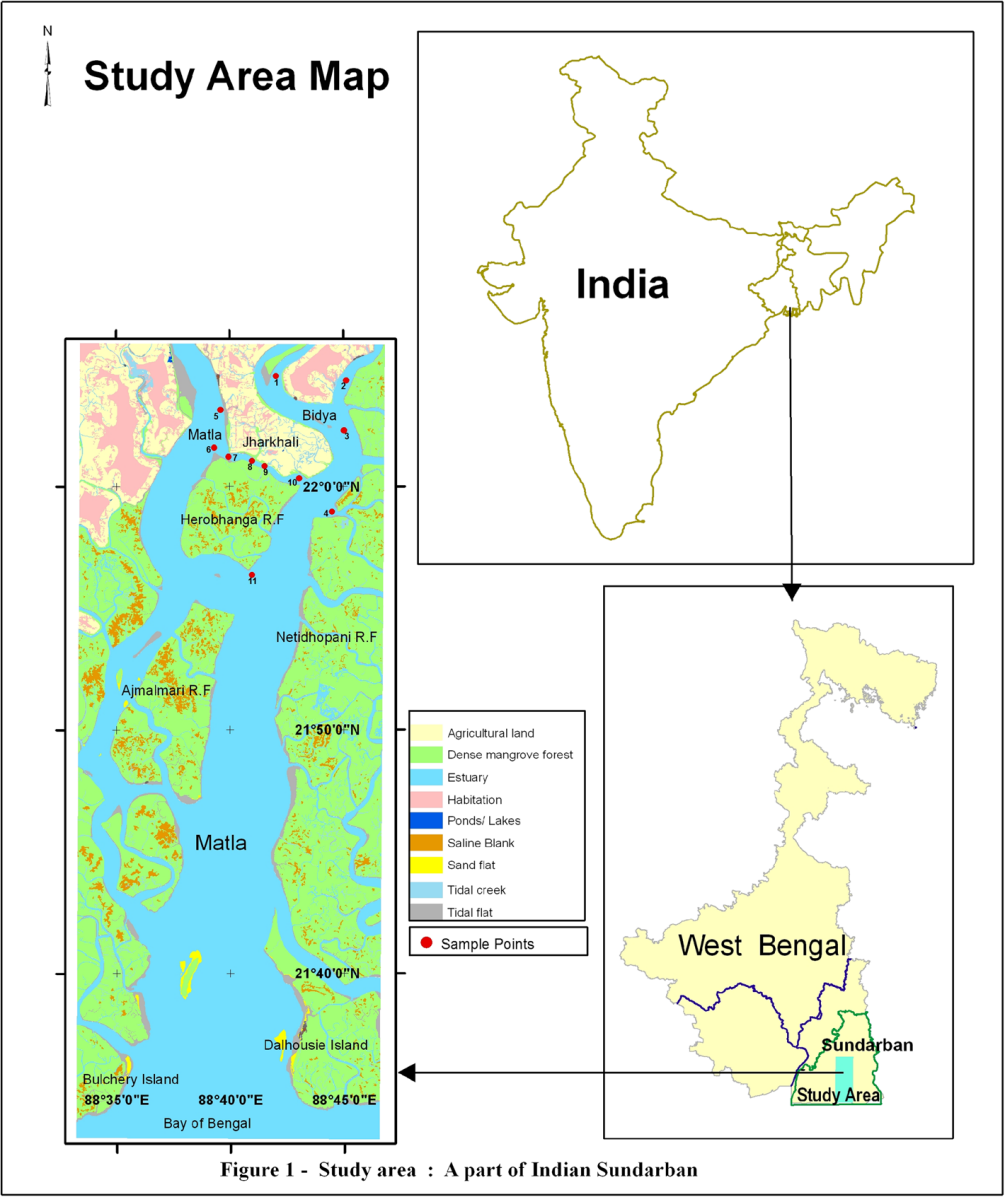
**Map of the Sundarbans showing sampling area** .

Our study area, Jharkhali estuary is a part of Hoogly-Matla estuary situated in Indian Sundarbans surrounded by the mangrove forests (Figure 1). This macrotidal estuary experiences a subtropical monsoon climate with annual rainfall of about 1600–1800 mm and is characterized by high tidal energy, moderate to high temperature (22°C - 34°C) and high nutrient influx.

Our study period consists of three seasons namely postmonsoon, premonsoon and monsoon. Postmonsoon period extends from November to February and is characterized by moderate to low temperature (21-27°C), moderate salinity (14–17 PSU) and intermediate aquatic turbidity (25–55 NTU) with little or no rainfall. Premonsoon period extends from March to June and is characterized by moderate to high temperature (27–34°C), high salinity (21–24.8 PSU) and low aquatic turbidity (20–25 NTU) with little or no rainfall but occasionally with premonsoon shower. Monsoon period extends from July to October and is characterised by high temperature (29-32°C), low salinity (12–15 PSU) and high aquatic turbidity (57–125 NTU) with moderate to heavy rainfall.

Present work reports the productivity of this subtropical estuary and explores its correlation with standing crop, nutrient concentration, turbidity, tidal fluctuation and tidal current velocity. Many other physicochemical parameters like temperature, conductivity, dissolved oxygen concentration (DO), sechhi depth were also monitored throughout the study period. Bacterial abundance was also estimated and its relationship with community respiration and nitrification rates was worked out. In a nutshell, this work explores the estuarine metabolism as a function of both physico-chemical and biological processes.

## Materials and method

### Study area

The study area includes Jharkhali Island in the north-central part. Two major channels namely Matla and Bidya run respectively on eastern and western sides of Jharkhali. These rivers are connected with each other by a prominent creek, locally known as ‘Bara Herobhanga Khal’ which is about 150 meters in width. Apart from this creek, several smaller creeks and rivulets present within the study area also act as conduits of water from both sides. ‘Herobhanga Reserve Forest’ is located just south of the Jharkhali Island. Both Bidya and Matla rivers are flanked by inhabited islands and mangrove islands.

Altogether 11 (eleven) sample points were selected (Table 1, Figure 1). Out of these eleven points, points 5 and 6 lie in Matla River and Points 1, 2, 3, 4 and 11 are within Bidya River, while points 7, 8, 9 and 10 lie within the Baro Herobhanga Khal (creek) joining these two mighty rivers. These points have been selected keeping in mind the hydrodynamics of the region.

**Table 1:** Details of the sample points in Sundarban estuary with specific latitude and longitude

**Details of Sample Points**	**Latitude**	**Longitude**
Point No. 1	22° 04' 13.42"	88° 41' 56.29"
Point No. 2	22° 04' 23.79"	88° 45' 03.48"
Point No. 3	22° 02' 12.82"	88° 44' 45.11"
Point No. 4	21° 59' 00.15"	88° 44' 21.99"
Point No. 5	22° 02' 49.17"	88° 39' 39.82"
Point No. 6	22° 01' 37.96"	88° 39' 13.55"
Point No. 7	22° 01' 25.60"	88° 39' 52.08"
Point No. 8	22° 01' 07.78"	88° 40' 55.80"
Point No. 9	22° 00' 41.28"	88° 41' 17.43"
Point No. 10	22° 00' 37.59"	88° 42' 55.04"
Point No. 11	21° 56' 19.61"	88° 40' 56.46"

### Sample collection

Study period extended from November’08 to October’11 postmonsoon (November-February), premonsoon (March-June) and monsoon (July-October). Samples were collected from the water surface (0.5m depth) of all the eleven stations along the Matla and Bidya river. Field trips were arranged at each fortnight and approximately sixty trips were conducted to collect the samples. Collected samples were filtered for the study of physicochemical and biological parameters. Samples were preserved in cold condition and transported to the laboratory within three hours of collection to analyze immediately.

### Physico-chemical analysis

A suit of environmental variables such as temperature, pH, conductivity, turbidity, light, tidal fluctuation, current speed, nutrients were measured along with primary productivity for three years study period in Sundarban estuary.

Water temperature (accuracy: ±0.3°C), pH (accuracy: ±0.01) and salinity (accuracy: ±0.5% of reading) were measured *in situ* with Hach Portable Meters (HQ40d) and turbidity was measured by using portable turbidity meter (Hach 2100P, Turbidity accuracy: ±0.1NTU). Dissolved oxygen (DO) concentration was determined according to Winkler method [27], nutrients like total nitrogen (TN), total phosphate (TP), silicate and ammonia were measured according to the standard methodology [28]. The light attenuation coefficient was evaluated by an empirically derived relationship of 1.57/ Secchi disc depth [3]. Subsamples for suspended load (SPM) were filtered through pre-weighed 0.45 pm Millipore filters. They were stored in small petri dishes, dried for 24 h at 60°C and reweighed. The difference between the two weights equaled the amount of suspended load in the water [29].

Tide measurement was performed by Valeport MIDAS WTR non directional tide gauge serial no. 34890 (Valeport, U.K). The MIDAS WTR Wave Recorder was used to prove Linear Wave Theory analysis method of measurement. It has high accuracy, piezo-resistive pressure sensors and a fast response PRT temperature sensor as standard. Current speed and direction was measured with Aanderra made Doppler Current Sensor 4420 Serial no. 282 Signal type CANbus.

### Biological analysis

#### Phytoplankton biomass (Chlorophyll-a)

Chlorophyll samples are drawn from all stations, with a maximum vertical spacing of 10 m through the chlorophyll maximum layer; at least one sample is always taken within 5 m of the maximum concentration. Chlorophyll samples are filtered through Whatman GF/F (0.45μ) filters and extracted in acetone in dark and refrigerated condition. Chlorophyll-a was determined spectrofluorimetrically [30].

#### Estimation of standing crop

Phytoplankton standing crop (biomass) was estimated by cell counting method. Phytoplankton were collected from surface water and preserved after treatment with Lugol’s iodine and buffered formaldehyde. Subsamples (1–2 liter) were used for quantitative enumeration utilizing a Sedgwick-Rafter counting chamber and Zeiss research microscope according to UNESCO PROTOCOL [31]. Total number of phytoplankton present in a litre of water sample was calculated using the following relation:

(1) $$N=\left(n\;x\;v\right)/V$$

Where, N = Total number of phytoplankton cells per litre of water filtered.

n: Average number of phytoplankton cells in 1 ml of plankton sample taken for counting.

v: Volume of plankton concentrate (ml)

V: Volume of total water filtered (l)

Standing crop/ m^3^ of water = N X 1000

Quantitative estimation of bacterial population was performed using Zeiss confocal fluorescence microscope. Bacterial cells were counted on black polycarbonate nucleopore membrane (pore size: 0.22 μm) using acridine orange stain [32,33].

#### Primary productivity

Primary productivity in a water body can be determined by different methods following the guidance of APHA [34]. We adopted the oxygen method where changes in oxygen concentration were measured in light and dark bottles. Planktonic photosynthesis rates were derived in terms of evolved oxygen in the process. These rates were converted into carbon units assuming photosynthetic quotient (PQ) of 1.3 based on C: N: P molar elemental composition of phytoplankton [35], and a respiratory quotient (RQ) of 1 [36]. The advantage of this method is that it estimates gross (GPP) and net (NPP) productivity along with community respiration (CR).

One of the sources of uncertainty in estimating gross primary production (GPP) is the metabolic activity of chemoautotrophic bacteria. This bacterial population is very active in estuaries and consumes significant quantity of oxygen in nitrification process. The oxygen consumption should be included to avoid overestimation of community respiration (CR). This problem was resolved by introducing one additional set of incubation containing nitrification (NR) inhibitor (Nitrapyrine and Chlorate, 5 mg/L and 10mM respectively) [37].

Samples were collected at each preselected depth (on the basis of light availability) in light bottles, dark bottles (with and without nitrification inhibitor) and in initial analysis bottles, each in triplicates. Light and dark bottles were suspended at the collection depth and incubated in the middle of the photic zone for 12 hours (from 6 AM to 6 PM). Dissolved oxygen of the initial bottles were fixed with NaI-NaOH and MnCl_2_ in the beginning of incubation period. At the end of the incubation period light and dark bottles were similarly fixed and all the bottles were brought back to laboratory in cold condition for analysis. Then dissolved oxygen concentrations were estimated by Winkler^’^s method [28].

In the dark bottles, amount of oxygen was reduced due to its consumption in respiration of plants, animals and bacteria, known as community respiration (CR). But in addition to community respiration, nitrification (NH_4_ – NO_3_) also consumed oxygen from water. Therefore, two metabolic processes i.e. respiration and nitrification simultaneously consume oxygen from water in the dark bottles. But in the dark bottle with nitrification inhibitor (NI), only respiration was responsible for oxygen consumption and therefore change in oxygen concentration provided the measure of community respiration (CR) only. In the dark bottle without nitrification inhibitor both respiration and nitrification were involved in oxygen consumption. So, the difference in oxygen concentration in the dark bottle with and without NI provided nitrification rate.

In the light bottle all the three metabolic processes namely photosynthesis (produced oxygen), respiration (consumed oxygen) and nitrification (consumed oxygen) occurred simultaneously. The difference in oxygen concentration in the light bottle provides net primary productivity (NPP). Gross primary productivity (GPP) is a measure of the sum of net primary productivity (NPP), community respiration (CR) and nitrification (NR). So, NPP, CR, NR and GPP can be estimated using the following relations:

(2) NPP=Light bottle–Initial bottle

(3) CR=Initial bottle–Dark bottle with nitrification inhibitor.

(4) NR=Dark bottle with nitrification inhibitor–Dark bottle without nitrification inhibitor

(5) GPP=Net primary productivity+community respiration+Nitrification rate.

#### Statistical treatment

During three years study period (November’2008 – October’2011) approximately sixty field trips were conducted (twice in a month). Samplings were made from eleven stations in triplicate and they were treated as subsamples. Reading of each month represented an average of 180 samples. The results were expressed as differences between the groups considered significant at p<0.05. Different statistical analysis and correlation regression analysis were performed using the software STATISTICA and in all cases p<0.01 indicating the relations were highly significant [38].

## Results

### Physicochemical parameters

Temperature of the estuary showed predicted range of variability indicating lowest in postmonsoon in the month of January (21.5°C) and highest in premonsoon in the month of June (33.5°C) (Table 2). The pH of water was weakly alkaline and more or less constant throughout the study period (8–8.15) (Table 2). The salinity of water increased gradually from postmonsoon (17.3 PSU) to premonsoon period (24.5 PSU) and decreased to a lowest value in monsoon (12.6 PSU). Highest salinity was observed in June (24.5 PSU) and lowest in October (12.6 PSU). Moderate to high DO concentration (6.5-9.8 mg/L) was observed throughout the year, highest in January (9.8 mg/L) and lowest in June (6.5 mg/L) (Table 2). Maximum aquatic turbidity was observed in monsoon in the month of October (125 NTU) followed by postmonsoon in the month of November (55 NTU) and premonsoon in the month of March (25 NTU), also evidenced by SPM concentrations (248.4 mg/L, 172.1 mg/L and 87.8 mg/L) respectively. This observation was also reflected in secchi disc indices (20.0cm, 52.3cm, 137.5cm) and light attenuation coefficients (0.078, 0.030, 0.011). Highest aquatic turbidity and SPM concentration were evidenced in October (125 NTU, 248.4 mg/L) and lowest in June (20 NTU, 57.5 mg/L) (Table 3). Nutrient concentration i.e. total nitrogen (TN), ammonia-nitrogen, total phosphate (TP) and silicate showed higher concentration in postmonsoon and monsoon compared to premonsoon (Figure 3a). TN and ammonia-nitrogen were estimated to be 34.14 μmol/L, 2.06 μmol/L in postmonsoon, 20.52 μmol/L,1.24 μmol/L in premonsoon and 28.22 μmol/L, 1.36 μmol/L in monsoon (averaged) respectively. Highest TN and ammonia-nitrogen concentration were observed in the month of February (36.25 μmol/L) and January (2.3 μmol/L) respectively and lowest in the month of June (14.15 μmol/L, 0.97 μmol/L). Total phosphate concentration was estimated to be 2.09 μmol/L in postmonsoon, 1.44 μmol/L in premonsoon, 1.73 μmol/L in monsoon (averaged), being highest in February (2.15 μmol/L) and lowest in June (1.08 μmol/L). Silicate concentration was observed to be 24.23 μmol/L in postmonsoon, 14.58 μmol/L in premonsoon and 22.47 μmol/L in monsoon (averaged) (Figure 3a). Highest silicate concentration was recorded in October (29.58 μmol/L) and lowest in June (11.02 μmol/L). TN:TP ratio was greater than Redfield ratio (16:1) in postmonsoon (averaged 17.3) and less than redfield ratio in premonsoon (averaged 14.5) and monsoon (averaged 14.7) (Figure 3b).

**Table 2:** Monthly variation of physical parameters in Sundarban estuary

**Month**	**Temperature (°C)**	**pH**	**Salinity (PSU)**	**DO (mg/L)**
November	27.5±0.1	7.90±0.1	14.9 ± 0.2	8.4±0.15
December	24.5±0.1	8.0±0.15	15.5 ± 0.1	8.5±0.12
January	21.5±0.1	8.10±0.1	16.6 ± 0.1	9.2±0.2
February	25.0±0.1	8.20±0.1	17.3 ± 0.1	9.8±0.18
March	27.5±0.1	8.10±0.12	21.2 ± 0.2	7.1±0.1
April	32.5±0.1	8.05±0.1	22.3 ± 0.2	7.0±0.12
May	33.5±0.1	7.95±0.14	23.6 ± 0.1	6.7±0.2
June	34.0±0.1	7.90±0.1	24.7 ± 0.1	6.5±0.15
July	33.5±0.1	7.95±0.15	15.0 ± 0.2	6.6±0.14
August	32.0±0.1	7.90±0.14	14.1 ± 0.1	6.9±0.12
September	30.5±0.1	7.90±0.1	13.3 ± 0.2	7.6±0.15
October	29.0±0.1	7.85±0.11	12.7 ± 0.1	7.8±0.18

Each value represents mean of 180 samples.

**Table 3:** Monthly variation of light availability parameters in Sundarban estuary

**Month**	**Suspended Particulate Matter (mg/L)**	**Turbidity (NTU)**	**Secchi Disc Index (cm)**	**Light Attenuation Coefficients**
November	172.1± 5.6	55±5.0	52.2±2.2	0.030
December	144.5± 4.2	48±5.5	61.5±2.1	0.025
January	129.4± 3.6	39±4.8	70.0±2.6	0.022
February	117.3± 4.6	25±5.3	78.0±1.8	0.020
March	87.8± 5.4	25±0.6	137.5±3.9	0.011
April	95.8± 5.6	24±0.4	169.6±4.2	0.009
May	73.4± 3.2	23±0.4	174.1±4.5	0.009
June	57.5± 6.2	20±0.8	185.3±5.6	0.008
July	186.5± 6.9	57±4.8	51.2±2.2	0.030
August	230.8± 2.7	110±3.2	24.5±1.1	0.060
September	234.2± 5.9	114±2.5	22.6±1.2	0.069
October	248.4± 6.9	125±6.2	20.0±1.1	0.078

Each value represents mean of 180 samples.

**Figure 2:**
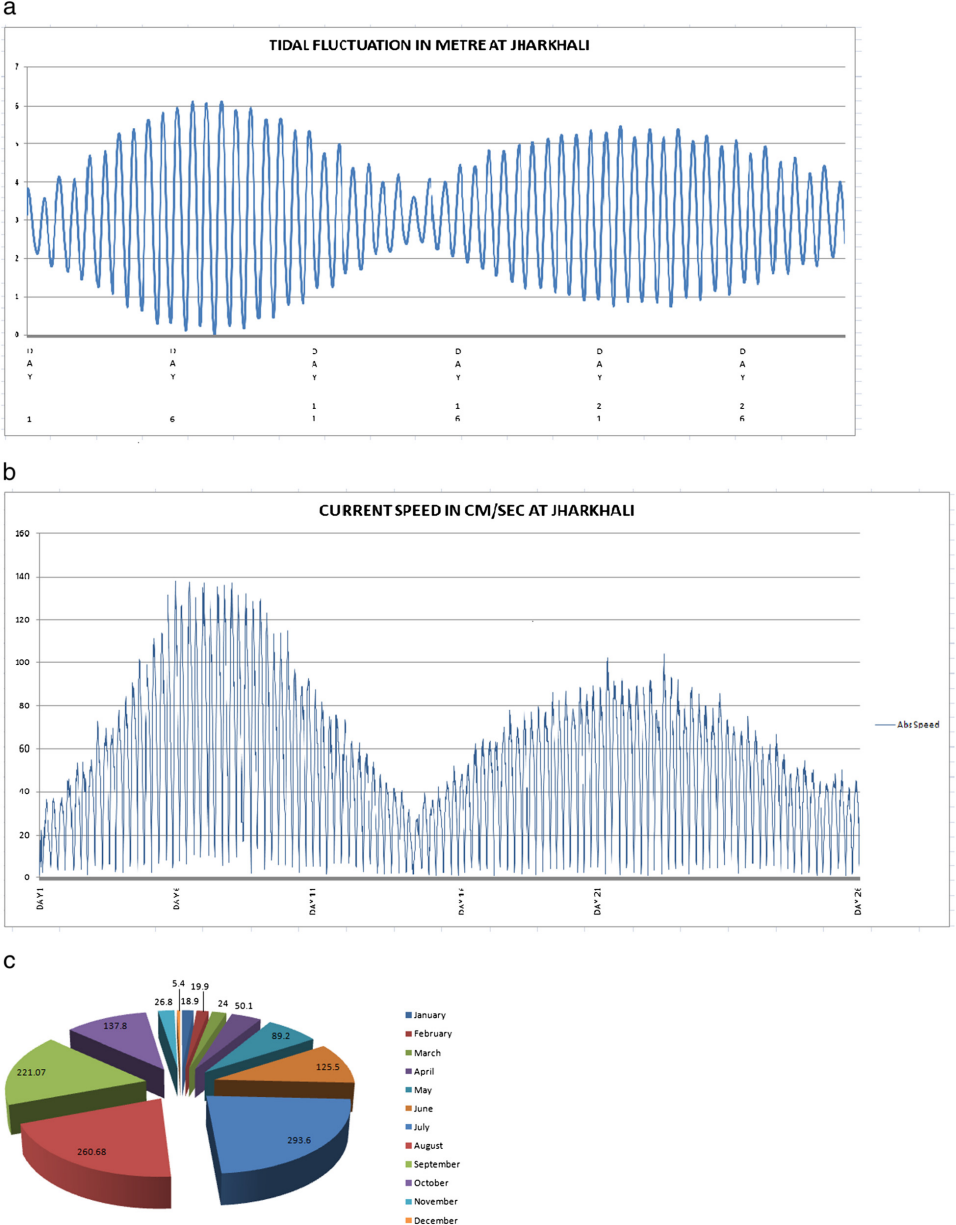
**a A representative profile of Tidal fluctuation at Jharkhali over the period of one month, showing mixed semidiurnal tidal pattern, with two consecutives spring tide nearly 15 days interval with a neap phase in between.** Maximum tidal range on Day 07 (6.1 metre) coincides with Blue moon event on Day 06. Subsequent highest peak on Day 22, having average tidal range 5.1metre corresponds to next new moon event. **b** Tidal current at Jharkhali for the period of one month, showing highest speed on Day 07 with velocity 140 cm/ sec, coinciding with maximum tidal range of Blue moon period and next highest speed attains on Day 22, matches on New moon event on the same day. **c** Distribution of monthly precipitation (mm) in Sundarbans. The precipitation data was averaged over 9 years (2002-2011) showing highest in July (21%) and lowest in December (0.4%).

**Figure 3:**
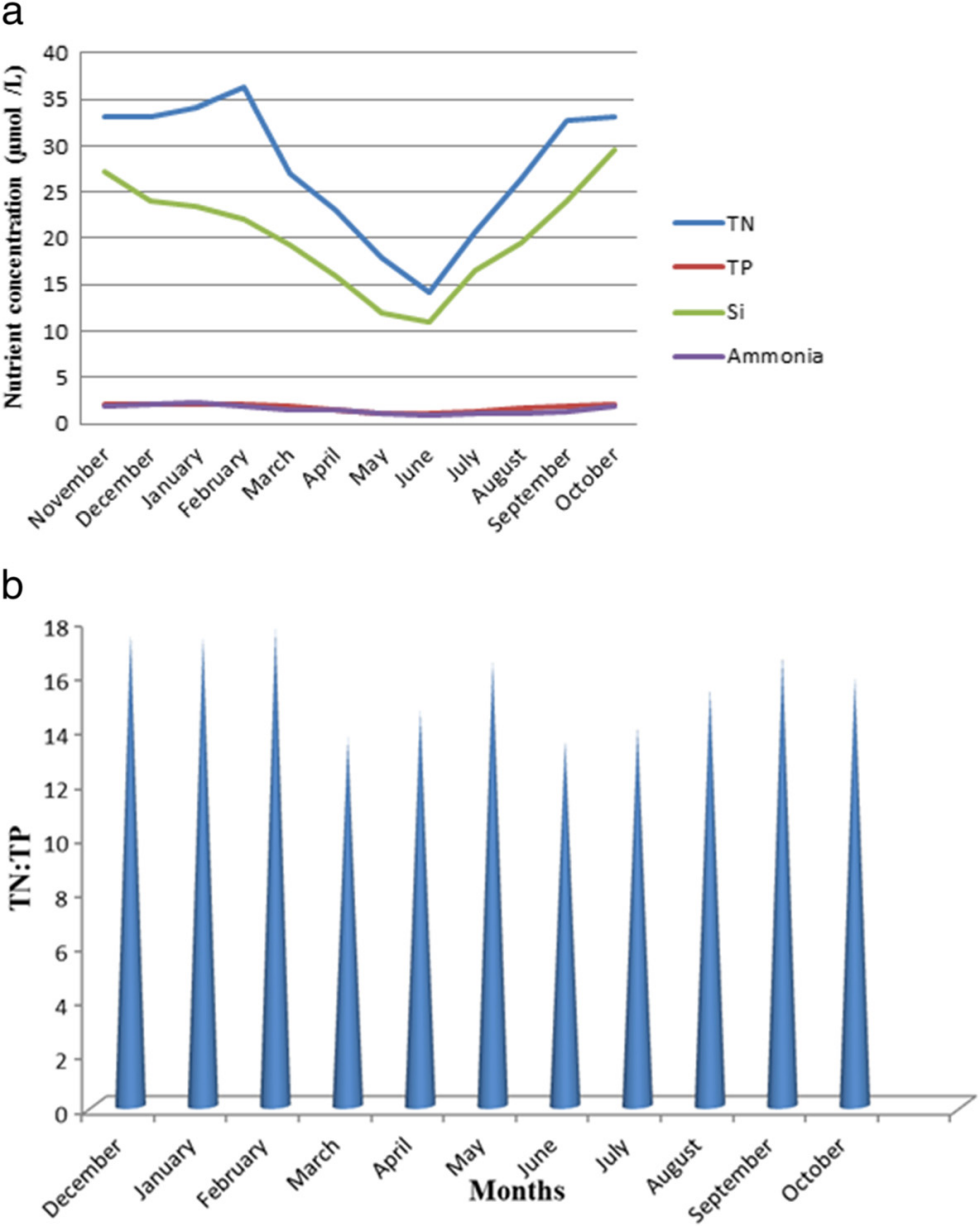
**a Monthly variation of nutrient concentrations such as total nitrogen (μmol/L), silicate (μmol/L), total phosphate (μmol/L) and ammonia (μmol/L) in Sundarbans estuary.** Each value represents mean of 180 samples. **b** Monthly variation of redfield ratio (Total Nitrogen/Total Phosphorus) ina Monthly variation of nutrient concentrations such as total nitrogen (μmol/L), silicate (μmol/L), total phosphate (μmol/L) and ammonia (μmol/L) in Sundarbans estuary Sundarbans estuary.

Tidal fluctuation ranged from 5.1 to 6 metre in height depending on spring to neap tide condition (Figure 3a). Tidal variation was observed to be quite high ranging from 5–6 metre in average and tidal wave travel from sea side which is nearly 50 km towards south to 30 km upstream to Port Canning. Current speed, which is the ultimate driving force for nutrient distribution in an estuary and it was observed to be quite high in this region, ranging from 140–180 cm /sec (Figure 2a and 2b). Sundarban estuary is characterized by subtropical monsoon climate with an annual rainfall of about 1600–1800 mm, maximum recorded in monsoon followed by premonsoon and postmonsoon (Figure 2c).

### Biological parameters

**Figure 4:**
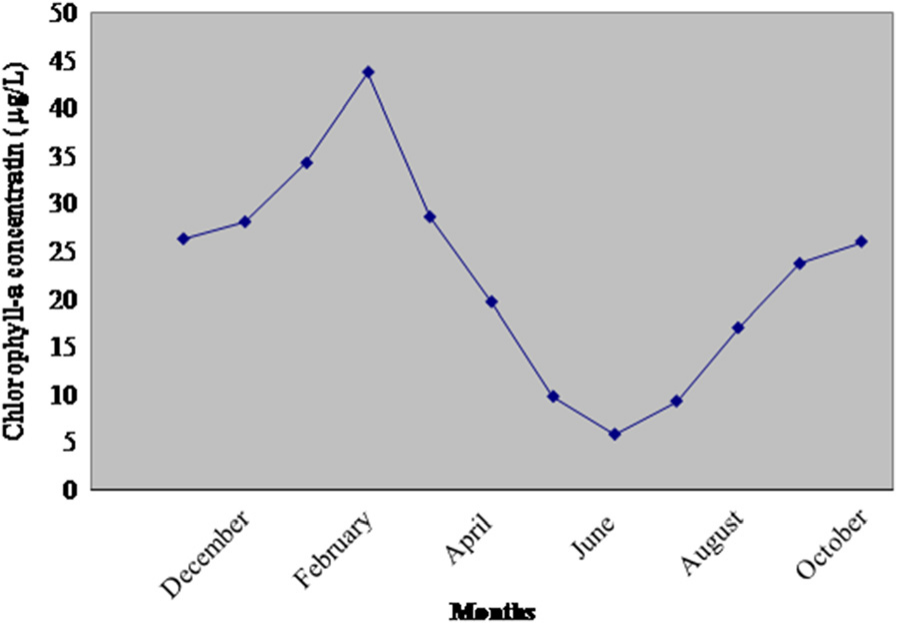
**Monthly variation of chlorophyll-a concentration (μg/L) in Sundarbans estuary.** Each value represents mean of 180 samples.

**Figure 5:**
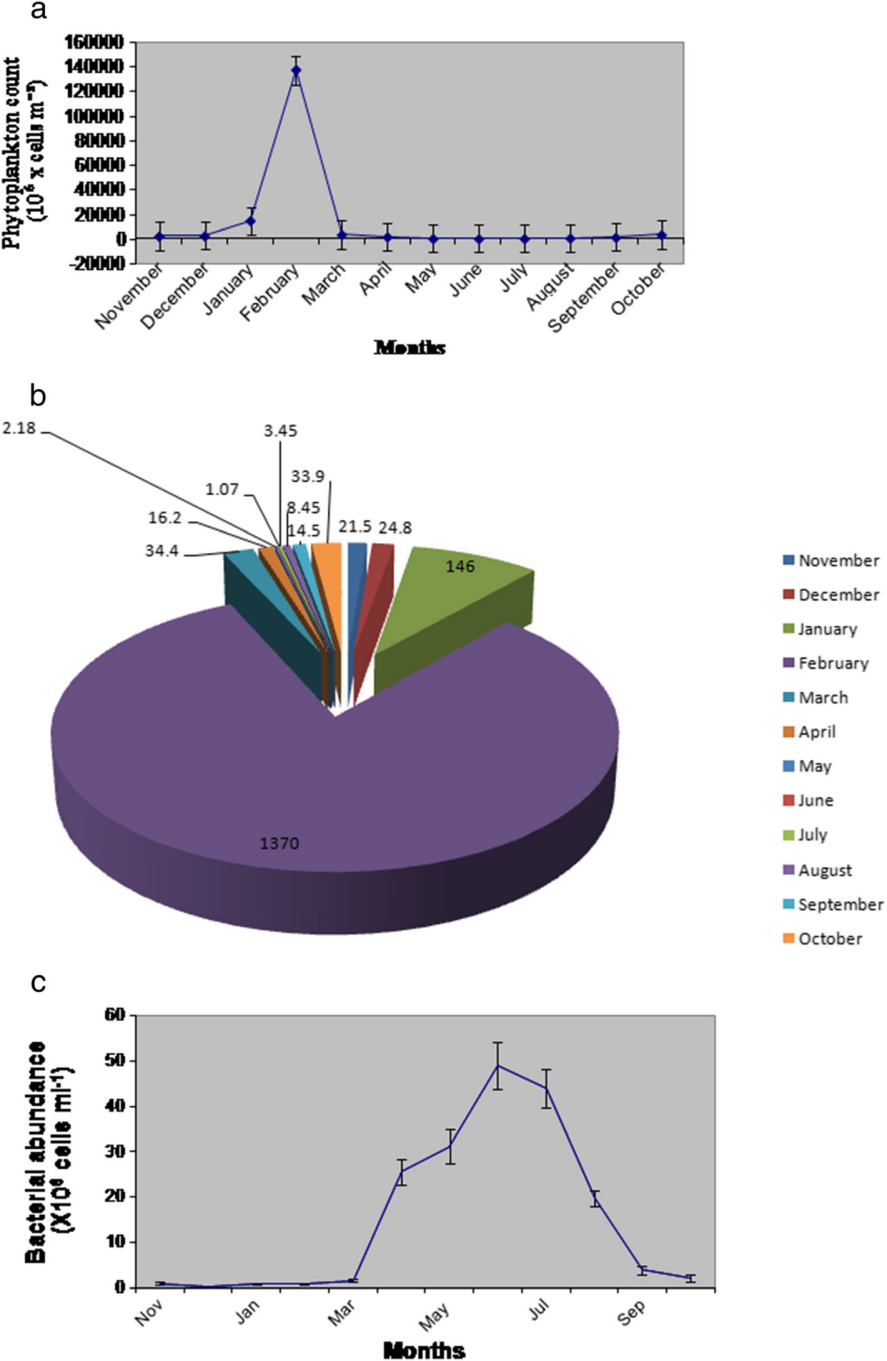
**a Monthly variation of phytoplankton standing crop (cells/m**^**3**^**) in Sundarbans estuary.** Each value represents mean of 180 samples. **b** Monthly variation of phytoplankton abundance (x 10^5^cells/L) in Sundarban estuary. Each value represents mean of 180 samples. **c** Monthly variation of bacterial abundance (cells/ ml) in Sundarbans estuary. Each value represent mean of 180 samples.

Annual cycle of phytoplankton biomass (chlorophyll-a concentration) and abundance (phytoplankton standing crop) evidenced seasonal variation being highest in February (43.80 μg L^-1^, 1.37 x10^11^ cells m^-3^ ) and lowest in June (4.85 μg L^-1^, 1.07 x10^8^ cells m^-3^ ) (Figure 4, Figure 5a and 5b).Chlorophyll-a concentration was observed to be 33.15 μg L^-1^ in postmonsoon , 16.02 μg L^-1^ in premonsoon and 19.05 μg L^-1^ in monsoon (averaged) (Figure 4). Phytoplankton standing crop ranged from 3.9X10^10^ cells m^-3^ in postmonsoon, 1.34X10^9^ cells m^-3^ in premonsoon and 1.39X10^9^ cells m^-3^ in monsoon (averaged) (Figure 5a and 5b). Bacterial abundance ranged from 5.14X10^5^ cells ml^-1^ in postmonsoon, 1.77X10^7^ cells ml^-1^ in monsoon and 2.68X10^7^ cells ml^-1^ in premonsoon (averaged), being the highest in June (4.9X10^7^ cells ml^-1^) and lowest in January (1.18 X 10^4^ cells ml^-1^) (Figure 5c).

**Table 4:** Gross primary production for major world estuaries and bays: a comparative assessment

**Estuary/Bay**	**Primary Production (g C m-2 y-1)**	**Referrences**
Dellware Estuary	307	Pennock and Sharp 1986 [47]
Chesapeake Bay	200	Cloern 1979 [48]
Hudson river estuary	180	Coljn 1983 [49]
**Sundarban estuary**	**151.07**	
Ems Dollard (middle) estuary	100 – 140	Coljn 1983 [49]
San Francisco Bay	130	Peterson 1979 [50]
Fraser river estuary	120	Parson et al. 1970 [51]
Columbia river estuary	90	Anderson 1975 [52]
Wassaw Sound estuary	90	Turner et al. 1979 [53]

**Figure 6:**
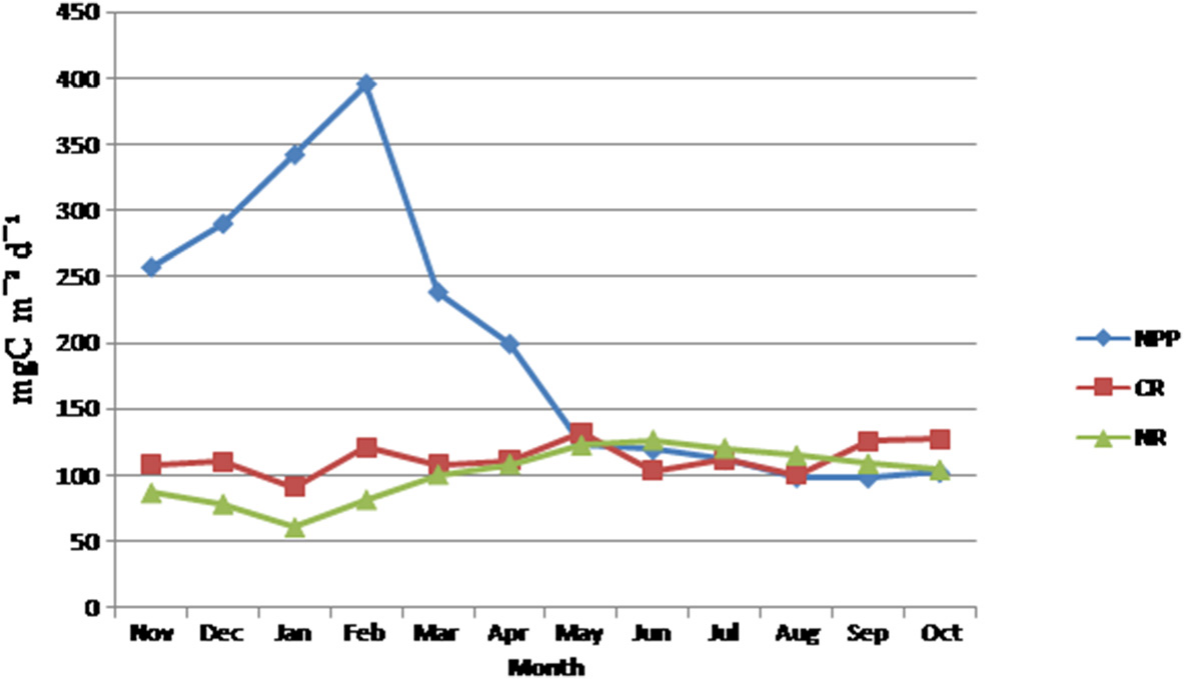
**Monthly variation of net primary production (NPP, mg C m**^**-2**^**d**^**-1**^**), community respiration (CR, mgC m**^**-2**^**d**^**-1**^**) and nitrification (NR, mgC m**^**-2**^**d**^**-1**^**) in Sundarbans estuary.** Each value represents mean of 180 samples.

**Figure 7:**
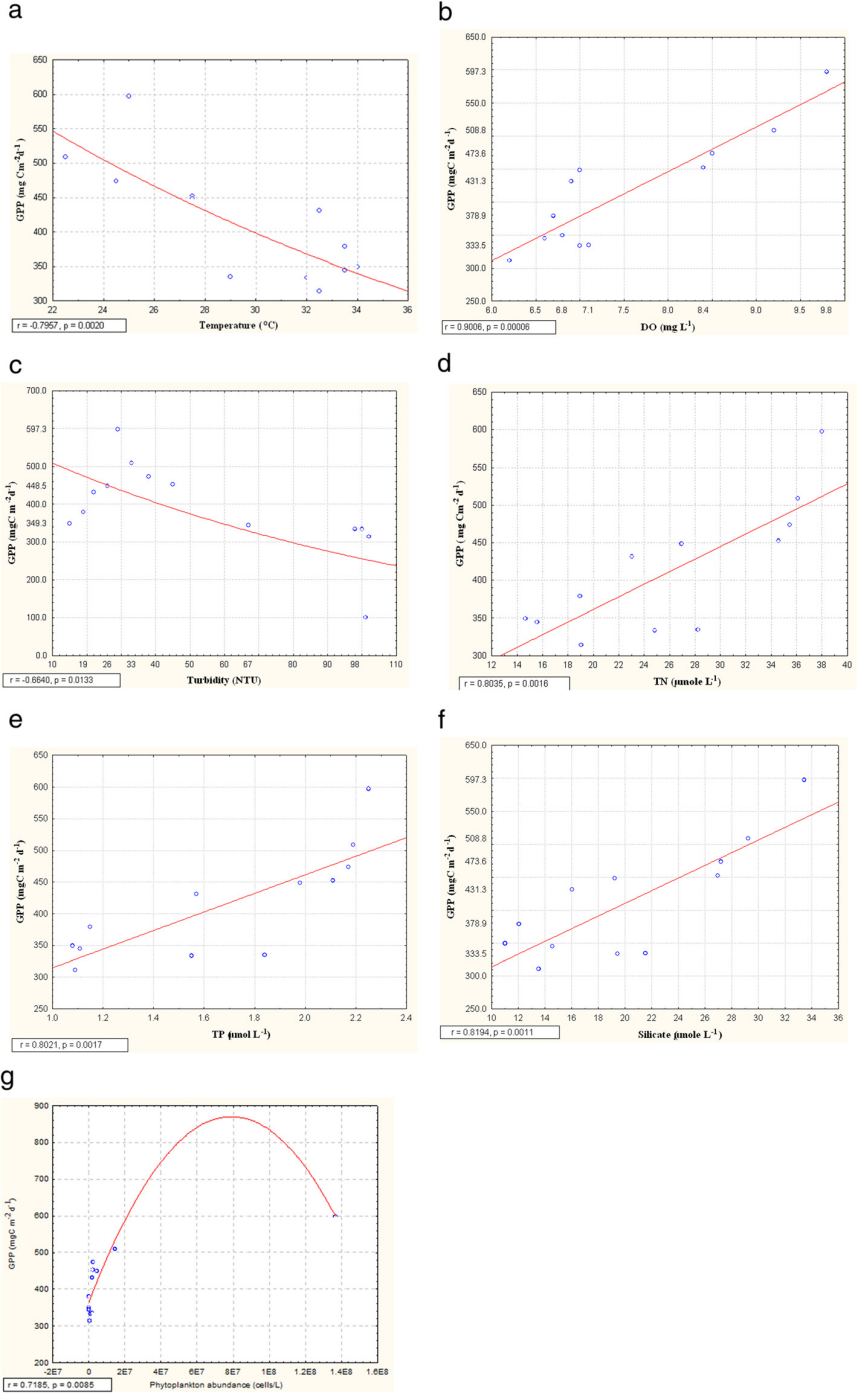
**Correlation of gross primary production (GPP, mg C m**^**-2**^**d**^**-1**^**) with ( a) temperature (°C) and (b) dissolved oxygen concentration (DO, mg/L), (c) turbidity (NTU), (d) total nitrogen (TN, μmol/L) (e) total phosphate (TP, μmol/L ), (f) silicate (μmol/L) and (g) phytoplankton abundance (cells/m**^**3**^**)** .

Phytoplankton primary productivity also followed a seasonal cycle being highest (597.3 mgC m^-2^ d^-1^) in the month of February (postmonsoon) and lowest (311.0 mgC m ^-2^ d^-1^) in the month of August (monsoon) (Figure 6). Primary productivity showed high degree of positive correlations with temperature (r=0.79, p=0.002) (Figure 7a), DO (r=0.90, p= 0.00008) (Figure 7b), TN (r=0.80, p=0.0016) (Figure 7d), TP (r=0.80, p=0.0017) (Figure 7e), silicate (r=0.81, p=0.0011) (Figure 7f) and phytoplankton standing crop (r=0.71, p=0.0095) (Figure 7g) and negative correlation with turbidity (r=0.66, p=0.013) (Figure 7c).

Photosynthesis depends on large number of external conditions, a regression equation was derived to describe the influence of various parameters like dissolved oxygen, turbidity, total nitrogen, total phosphate silicate and ammonia on NPP.

(6) $$\text{NPP}=-239.76+39.04\left[\text{DO}\right]-1.07\left[\text{TUR}\right]-11.21\left[\text{TN}\right]+149.96\;\left[\text{TP}\right]-25.41\left[\text{AMMONIA}\right]+14.19\left[\text{SILICATE}\right]$$

Where

R^2^ = 0.85 and

NPP = Net primary productivity (mgC m^-2^ d^-1^)

DO = concentration of dissolved oxygen (mg L^-1^)

Tur = turbidity (NTU)

TN = concentration of total nitrogen (μM)

TP = concentration of total phosphate (μM)

The coefficient of determination (R^2^ = 0.85) was relatively high and the relationship described by this equation was highly significant (p < 0.0001).

The pH and salinity level of water were not included in this equation as no significant correlations were detected between them and NPP. There were more than 500 observations in the data sets that include NPP for possible use in the equation. A sensitivity test was performed to identify the most dominant parameters. According to their importance the parameters can be organized in descending order: total phosphate> DO> silicate> turbidity> total nitrogen> ammonia.

**Figure 8:**
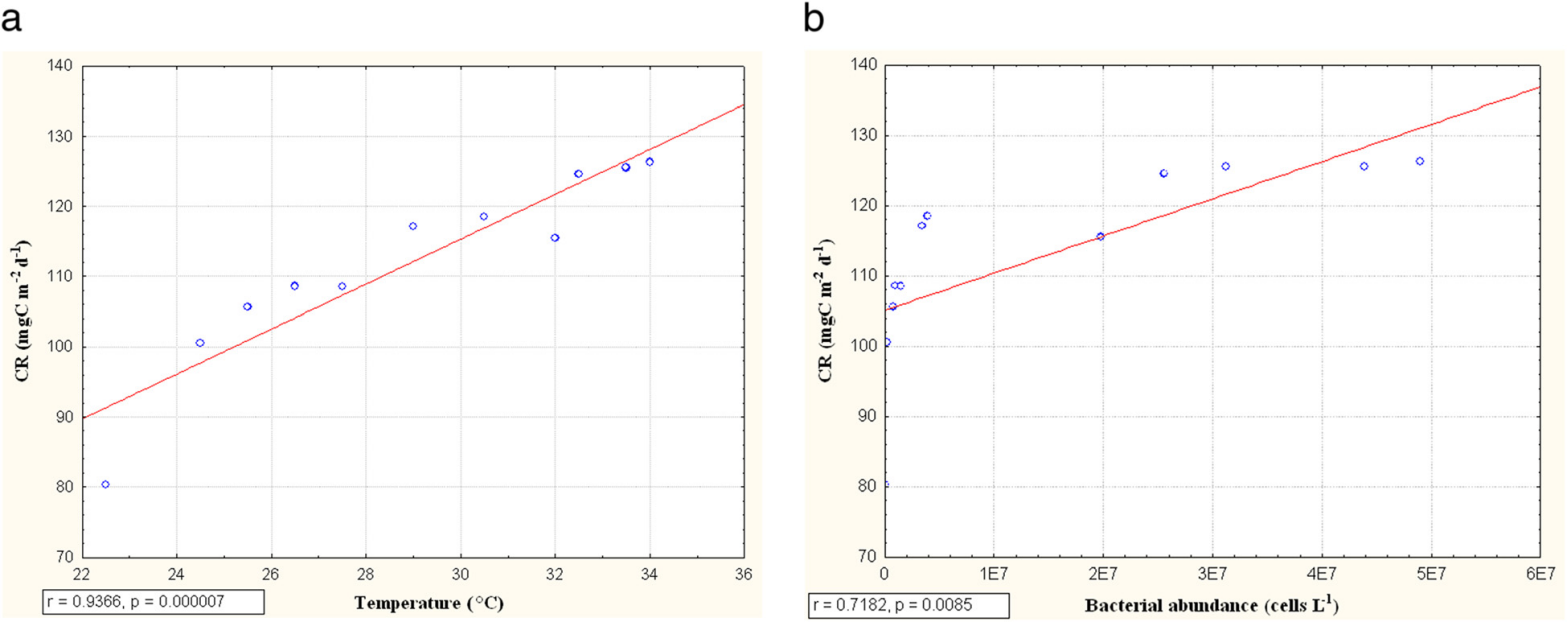
**Correlation of community respiration (CR, mgC m**^**-2**^**d**^**-1**^**) with (a) temperature (°C) and( b) bacterial abundance (cells/ ml)** .

**Figure 9:**
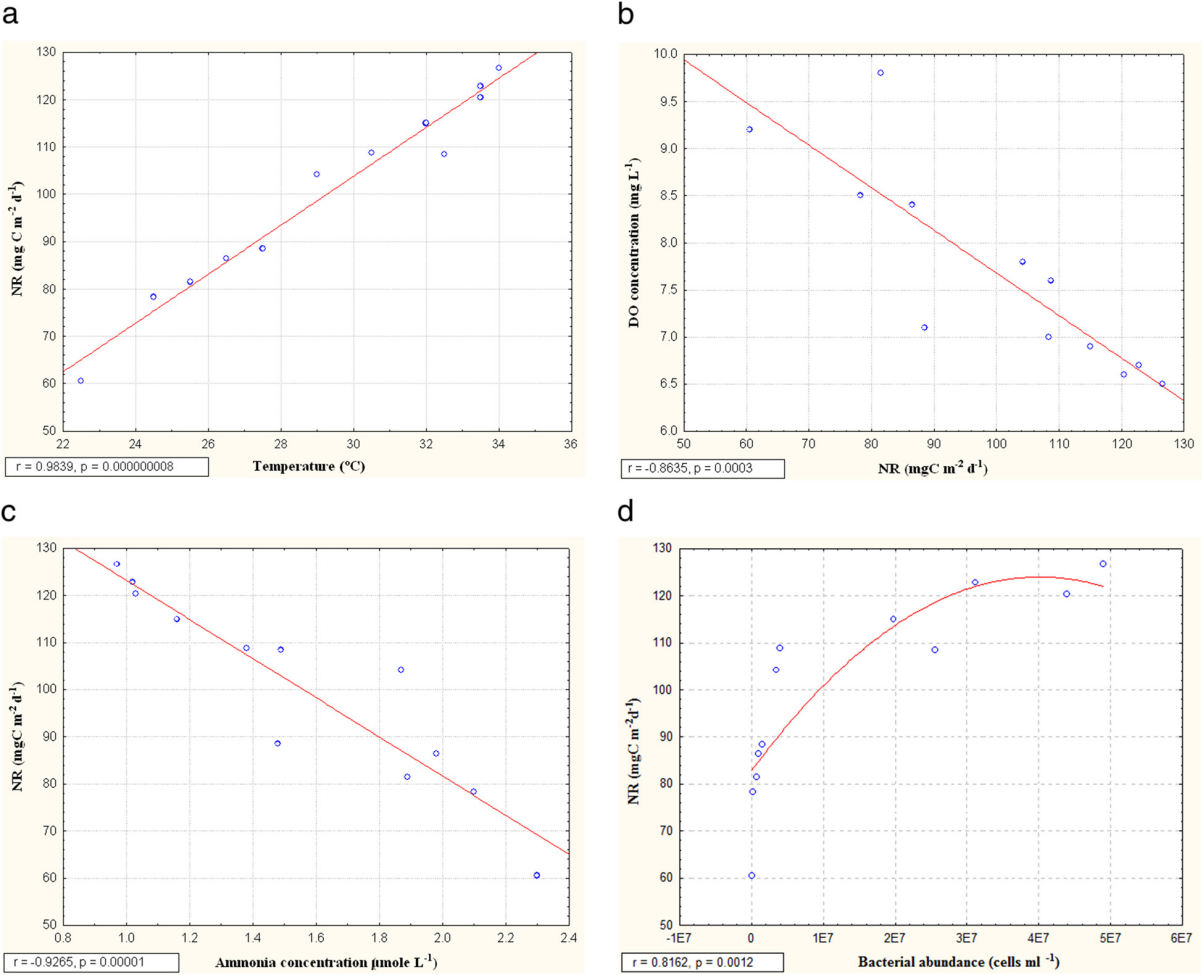
**Correlation of nitrification (NR) with (a) temperature (°C) (b) dissolved oxygen concentration (DO, mg/L), (c) ammonia concentration (μmol/L) and (d) bacterial abundance (cells/ml)** .

**Figure 10:**
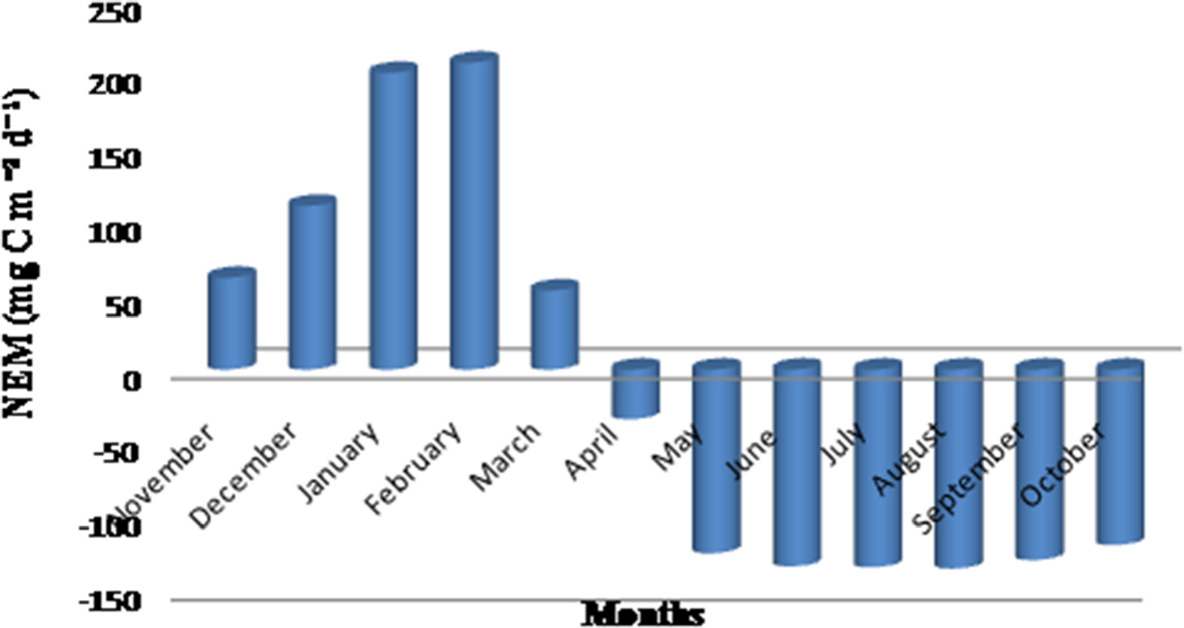
**Net ecosystem metabolism in Sundarban estuary** .

Community respiration and nitrification rates were found to be the highest in premonsoon in the month of June (126.3 mgC m^2^ d^-1^ and 126.6 mgC m^2^ d^-1^) and lowest in postmonsoon in the month of January (80.3 mgC m^2^ d^-1^ and 60.5 mgC m^2^ d^-1^) for Sundarban estuary (Figure 6). Community respiration and nitrification rates indicated positive correlations with temperature (r= 0.94, p=0.000007 and r=0.98, p=0.000000008) (Figure 8a and Figure 9a). Both community respiration and nitrification evidenced positive correlations with bacterial abundance (r=0.72, p=0.009 and r=0.82, p=0.0012) (Figure 8b and Figure 9d). Nitrification showed negative correlations with DO and ammonia concentration (r=0.86, p=0.0003 and r= 0.93, p =0.00001) (Figure 9b and 9c). There was a seasonal shifting between autotrophic and heterotrophic conditions in the estuary, net autotrophic in postmonsoon and net heterotrophic in premonsoon and monsoon (Figure 10).

## Discussion

Estuaries are dynamic ecosystems, forming a transition zone between river and ocean environments. They are subject to both marine influence, such as tides, waves and the influx of saline water and other riverine influences. The primary productivity of an ecosystem also depends on the favorable status of the physical characteristics and hydrodynamic conditions of the estuary [39]. Phytoplankton species composition, richness, population density, and primary productivity will vary from coast to coast and sea to sea depending upon the varying hydro biological features [40]. Our work expresses the metabolism of Sundarbans estuary as a function of physico-chemical and biological processes.

Gross primary production (GPP) predictability evidenced high degree of positive correlation with phytoplankton abundance and standing crop i.e. biomass. This is expected as higher level of plankton biomass enhances the sites for photosynthesis. A moderate degree of correlation was observed between GPP and temperature as indicated by a negative slope. GPP was found to be declined above an optimal temperature of 25.5°C and it was also positively correlated with DO level of water. Fairly high concentration of DO throughout the year indicated consistently high wave action (Figure 2a and 2b) and steady state primary productivity of the estuary. Turbidity of water influenced the primary productivity through its effect on light attenuation indicating negative exponential correlation with gross primary production [41]. The pH value of water did not bear any significant correlation with gross primary production and remained almost constant throughout the year. This is contrary to general notion that removal of CO_2_ from water during photosynthesis enhances the pH value [42]. Primary productivity increased steadily from November to February, despite the fact that salinity of water was increased during the study period. Thus, primary production was independent of salinity as long as the salinity regime did not exceed a certain tolerance level. But this pattern broke down at high salinity values in the month of April. At salinity level 24.7 PSU primary productions as well as planktonic biomass was reduced indicating that the threshold tolerance level of majority of planktonic species was crossed [38] in the estuary.

The tidal fluctuation is the main driving force behind distribution of nutrients along the entire estuary. The distribution pattern and dispersion of the nutrients and pollutants within the estuary is dependent on the different motions of the water body. In estuaries, nutrient availability is generally adequate to support primary productivity [43]. Gross primary production bore high degree of positive correlation with total nitrogen concentration. Similarly, primary productivity was positively correlated with total phosphate content. Highest phytoplankton standing crop (biomass) and productivity was observed during post monsoon and magnitude of these maxima was closely related to nitrogen and phosphorus availability in the aquatic system. These findings were in good agreement with Boynton et al. [14]. This estuary was characterized by high level of total nitrogen and moderate to low level of total phosphate (Figure 2). High leaf litter loading from surrounding mangrove forest was the possible reason for nitrogen enrichment of the estuary. Primary growth limiting nutrient for phytoplankton appeared to be predictable from total nitrogen: total phosphorous (TN:TP) ratios [44]. During postmonsoon TN:TP ratio was greater than Redfield ratio (16:1), so the estuary was phosphorus- limited in this time whereas it was nitrogen-limited during monsoon and pre monsoon (Figure 3b) since TN:TP value was less than Redfield ratio [44-46]. Silicate concentration was observed to be higher throughout the year (Figure 3a), the highest silicate concentration being recorded in monsoon (October) and late monsoon (November). Silicate is a primary growth limiting nutrient for diatoms. Silicate concentration was found to be highest during monsoon and late monsoon due to run-off and seasonal opening of upstream channels of Sundarbans. Silicate concentration also evidenced a positive correlation with primary productivity.

Phytoplankton production in the estuary displayed marked seasonal variability, lowest value being recorded in August (monsoon) (314.0 mg m^-2^d^-1^) and highest in February (postmonsoon) (597.3 mg m^-2^d^-1^) with an average of 151.07 gC m^-2^ Y^-1^ compared to other nutrient rich estuarine estuaries of the world (Table 4) [47-53]. The NPP of the estuary reached its peak in the post monsoon months. An examination of physical and chemical parameters revealed that most of them stay at a “comfort-zone” favouring high level of photosynthesis during this time. Total nitrogen, phosphate and silicate levels of estuarine water reached their maximum level, making the estuarine water nutrient rich for metabolic activity. This nutrient enrichment of water was achieved by land mass wash off from adjoining agricultural lands, drainage of waste from shrimp culture farms and upwelling of sediments during monsoon. Among physical factors, light penetration and temperature remained conducive for photosynthesis. The cyclonic activity died down during this time allowing estuarine water to become calm and clean, thereby allowing greater light penetration. The temperature also remained at the optimal level.

At the premonsoon season, this favourable condition for high productivity gets disturbed due to drastic change in a few parameters. The dominant parameters adversely affecting NPP were high temperature and high salinity. As a result, the estuary showed marked decline in NPP.

During monsoon months, the turbidity of water became severely limiting due to high suspended load to block light penetration towards sub-surface levels. High nutrient concentration and favourable conductivity level or high DO of water could hardly help in elevating the NPP value.

The community respiration (CR) of the estuary ranged from 91.35 mgC m^-2^d^-1^ to 132.3 mgC m^-2^d^-1^. The CR value was found to be highest in the month of June and lowest in January. The CR value is observed to be correlated positively with temperature and bacterial abundance. These findings are in agreement with Iriarte et. al. [54] for a temperate estuary in Northern Spain. These high rates of CR in summer months in the estuary must be associated to enhanced organic carbon input from sources other than the primary production since the later was low during summer. Fresh water flow cannot explain this unknown carbon source to the estuary as there was hardly any rainfall in summer and connection to fresh water river is cut off due to siltation. Thus high CR value during summer may only be explained by an increase of lateral organic carbon input into the estuary possibly from mangrove vegetation in the catchment. This organic carbon consumed and stored into the sediments probably balances the carbon budget of the estuary [55]. Thus planktonic CR in the Sundarbans estuary was primarily driven by allochtonous organic matter rather than local production. Similar feature was also observed in other estuaries of the world e.g. Scheldt estuary [56].

The nitrification rate (NR) of the estuary ranged from 60.5 mg C m^-2^ d^-1^ to 123.6 mg C m^-2^ d^-1^, attaining the highest in June and lowest in January indicating a positive correlation with ambient temperature [54]. This can be explained on the basis of bacterial abundance in the estuary showing a positive correlation with NR. During the January-June period ammonia-nitrogen content also declined steadily, revealing a negative correlation with NR. Since ammonia is the substrate on which nitrifying bacteria metabolize, the observed pattern is self explanatory. Reduced nitrification level should ideally improve the DO level of water. Elevated DO level in response to decreased nitrification activity in postmonsoon and vice versa in premonsoon establishes this hypothesis i.e. DO level is negatively correlated with nitrification activity.

Estuaries function as a ‘biogeochemical reactor” since many autochthonous processes occur here [57,58]. The metabolic state of an ecosystem maintains a dynamic balance between primary production and community respiration [26]. This estuary remained autotrophic for five months (November to March) of the year. During this time the primary production is greater than community respiration resulting in export or burial of organic matter through conversion of inorganic matter and carbon dioxide. The estuary remained heterotrophic during remaining seven months (April to October). Community respiration was greater than primary production during that period and allochthonous materials are re-mineralized leading to production of inorganic nutrients and carbon dioxide. Thus Sundarbans estuary was a net sink of CO_2_ for five months of the year and net source of CO_2_ for remaining seven months. If net ecosystem metabolism of the entire year was taken as a whole, the estuary can be designated as a net source of CO_2_[22-24,59].

Trophic state of the estuary was worked out using chlorophyll-a concentration (Jones and Fredly, 1982). This estuary showed chlorophyll-a concentration greater than 10 μg/L throughout the year , except for the month of May and June (9.07 and 4.85 μg/L respectively). Thus, the estuary was identified as mesotrophic only in the months of May and June but eutrophic in the remaining ten months [38,60].

## Conclusion

Phytoplankton production in the tidal creeks in Sundarbans estuary averaged 151.07 g C m^-2^ Y^-1^ designating this ecosystem to be moderately productive compared to other nutrient rich estuarine ecosystems of the world (Table 4). Different indices and limiting relations achieved from the experiments support the generalized concepts regarding light availability, nutrient load, conductivity and bacterial abundance in an ecosystem. Average primary production was a function of nutrient loading and light penetration in the water column. This result was the outcome of a nutrient rich ecosystem which also potentiated the growth of phytoplankton. High aquatic turbidity, conductivity and suspended particulate matter were also the other limiting factors to attenuate light penetration with negative influence on primary production. The growth rate of phytoplankton populations was a function of light availability and the variability in biomass can be justified by variation of light exposure. Community respiration and nitrification rates were also influenced by the bacterial abundance in the estuary. Sundarban estuary was identified as a tropical, well mixed (due to high tidal influx) and marine dominated (as there is no fresh water connection) system.

## Competing interests

The authors declare that they have no competing interests.

## Authors’ contributions

KC and SM performed all experiments, calculated results, prepared the tables, graphs and diagrams, and composed the draft manuscript in consultation with MB. MB designed the experiments, analyzed and interpreted data and results, modified the manuscript in the final form. KSS prepared the map and performed the tide and current experiments. PN participated in the field experiments and assisted in preparing manuscript. SB planned the project, was involved in acquisition of funds, selected the site and field stations including the geo-referencing of the stations, prepared the map, guided the field study, sample collection and the tide and current experiments. All authors read and approved the final manuscript.
